# Tai Chi for the prophylaxis of episodic migraine: protocol of a non-inferiority randomized controlled trial with mechanism exploration

**DOI:** 10.1186/s12906-023-04154-x

**Published:** 2023-09-18

**Authors:** Yao Jie Xie, Xiaoli Liao, Stanley Sai-chuen Hui, Longben Tian, Wing Fai Yeung, Alexander Yuk-lun Lau, Stefanos Tyrovolas, Yang Gao, Xiangyan Chen

**Affiliations:** 1 School of Nursing, Faculty of Health and Social Sciences, The Hong Kong Polytechnic University, Hong Kong, Hong Kong SAR China; 2 Research Centre for Chinese Medicine Innovation, The Hong Kong Polytechnic University, Hong Kong, Hong Kong SAR China; 3 Department of Sports Science and Physical Education, The Chinese University of Hong Kong, Hong Kong, Hong Kong SAR China; 4 Department of Medicine and Therapeutics, Faculty of Medicine, The Chinese University of Hong Kong, Hong Kong, Hong Kong SAR China; 5 Department of Sport, Physical Education and Health, Hong Kong Baptist University, Hong Kong, Hong Kong SAR China; 6 Department of Health Technology and Informatics, Faculty of Health and Social Sciences, The Hong Kong Polytechnic University, Hong Kong, Hong Kong SAR China

**Keywords:** Migraine, Tai Chi, Women, Randomized controlled trial

## Abstract

**Background:**

Migraine is a complex neurovascular disorder with considerable clinical, social and economic issues. Tai chi has the potential to be an alternative prophylactic treatment for migraine with high safety since the adverse effects and limited efficacy of available medications.

**Aims:**

The proposed study aims to compare the prophylaxis efficacy of 24-week Tai Chi training on migraine attacks with the standard prophylactic medication; and to explore the mechanism of Tai Chi in preventing migraine attacks by analyzing the associations between changes of migraine attacks and changes of neurovascular functions and inflammatory makers.

**Method:**

This is a two-arm parallel non-inferiority randomized controlled trial. In total 220 Hong Kong Chinese women aged 18–65 years with diagnosis of episodic migraine will be recruited and randomized to either the Tai Chi training group or the standard prophylactic medication group with 1:1 ratio, and receive the 24 weeks of modified 33-short form Yang-style Tai Chi training and the standard prophylactic medications, respectively. A 24-week follow-up will be implemented for both groups. For efficacy examination, the primary outcome was the frequency of migraine attacks measured by the migraine diary; and for the mechanism exploration, the primary outcome was the volume and number of white matter hyperintensity (WMH) measured by magnetic resonance imaging (MRI). The measurements will be conducted at the baseline, 24th weeks, and 48th weeks. Linear mixed model will be adopted to comprehensively analyze the changes of variables within and between groups.

**Discussion:**

Given the importance of reducing disease burden and financial cost of migraine attacks, the findings of this study will provide new insights regarding the role of Tai Chi in alleviating migraine burden and further shed light on the mechanism action of Tai Chi on preventing headache attacks.

**Trial registration:**

ClinicalTrials.gov NCT05690737. Registered on January 28, 2023.

## Introduction

Migraine is a chronic neurovascular disorder featured of episodic headache attacks, which is the most common primary headache disorder and the second most prevalent neurovascular disorder worldwide [1, 2]. Migraine can be divided into episodic or chronic form based on the number of headache days per month, episodic migraine refers to a diagnosis of migraine with a headache pattern of fewer than 15 days per month in the last 3 months [3]. Episodic migraine accounts for more than 90% of migraine cases and can progress into chronic migraine at a rate of 2.5% per year [4]. Migraine presented a global prevalence of 14.4%, with a female prevalence of 18.9% and a male prevalence of 9.8% in the Global Burden of Disease Study 2016 [1]. Migraine led to 45.1 million years lived with disability (YLD) globally, which was the first global cause of YLD in young women and the second cause of YLD in both gender [5]. The biological basis and pathogenic mechanism of migraine remain elusive, evidence supports the combined involvement of neuropathological, cerebrovascular and inflammatory aspects. Migraine is a public health concern with considerable clinical, social and economic burden, which urgent development of prophylaxis measures to alleviate physiological pain, mobility, discomfort and health cost [6].

Both pharmacological and non-pharmacological treatments play a role in the prophylaxis of migraine [7]. Pharmacological prophylactic treatments, like topiramate, beta blockers, or antidepressants, have been recommended for migraineurs who have more than two headache attacks per month. While many migraineurs benefit in some aspects from pharmacological prophylactic treatments, they also interference with low tolerability, overuse syndromes and rebound headaches [8]. In contrast, non-pharmacological prophylactic treatments appear to be relatively safe with both high tolerability and patient satisfaction. Tai chi, a traditional Chinese exercise derived from martial arts folk traditions, has been broadly applied as alternative treatment for many neurological and psychological disorders [9]. Clinical evidence converges to support the prophylactic role of aerobic exercise on migraine, as aerobic exercise exhibits the potential to reduce the frequency, duration and intensity of migraine attacks in clinical trials [10]. Tai chi, as a form of aerobic exercise, shares the health potentials with other aerobic exercises to improve neurochemical factors and cerebrovascular fitness [11]. Tai Chi has been used in the treatment of general headache. A randomized controlled trial conducted among 47 adult patients with tension headache found that the 15-week Tai Chi program effectively relieved headache, improved fatigue status and energy expenditure in patients with tension headache [12]. A pilot randomized controlled trial (RCT) from our research team preliminarily supported the effect of Tai Chi training on migraine prophylaxis [13]. This pilot RCT was conducted among 73 women with episodic migraine, which found that a 12-week Yang-style Tai Chi program could significantly reduce 3.0 times and 3.6 days of migraine attacks per month after the intervention [13]. Tai Chi not only exhibited comparable clinical effects with some well-established non-pharmacological prophylactic treatments like relaxation training [14], but also displayed similar clinical effects as prophylactic medications in this pilot study [13].

The pathophysiology of migraine attack is believed to be highly complex involving neuropathological, cerebrovascular and inflammatory mechanisms. A recent meta-analysis found a higher prevalence of white matter hyperintensity (WMHs) in migraineurs as compared to age-matched controls, which supported the involvement of changes in brain structure in the neuropathological of migraine [15]. Cerebrovascular mechanism also plays a role in the occurrence of migraine attacks. Migraineurs have altered cerebrovascular function, as represented by elevated pulsatility index (PI), higher resting mean blood flow velocity (MBFV), and impaired cerebrovascular responsiveness (CVR) to hypercapnia [16]. Regard to inflammatory mechanism, inflammatory mediators contribute to the activation of the trigeminal system in a manner may drive the migraine headache [17]. The elevation of certain inflammatory markers has been observed in migraineurs [10]. Evidence indicated that Tai Chi hold the potential to change white matter structures [18], improve cerebrovascular function [19] and regulate inflammatory responses [20]. It can therefore be inferred that Tai Chi may exert prophylactic effects through migraine pathogenesis in terms of neuropathological, cerebrovascular and inflammatory mechanisms. Nevertheless, the mechanism needs further essential and deeper exploration.

Further study with larger sample size and longer observation period should be performed to validate the clinical efficacy of Tai Chi by comparing with prophylactic medication and to explore its prophylactic mechanism. A two-arm randomized controlled trial with larger sample size and longer study period is thereby designed by the authors to investigate the clinical efficacy and mechanism action of Tai Chi on migraine prophylaxis.

### Objectives

The proposed study contains two objectives: efficacy examination and mechanism exploration.

- To determine the clinical efficacy of 24-week Tai Chi training in the prophylaxis of episodic migraine by comparing with standard prophylactic medication among Hong Kong Chinese women; and examine the prolonged effectiveness of Tai Chi on the migraine features, related disability, physical and mental health by additional 24 weeks follow-up.
- To explore underlying mechanisms of Tai Chi in the prophylaxis of migraine in three typical aspects: (1) white matter hyperintensity (WMH); (2) cerebral vasculature activity and cerebral blood flow velocity; and (3) inflammatory markers.

## Methods/design

### Study design

**Fig. 1:**
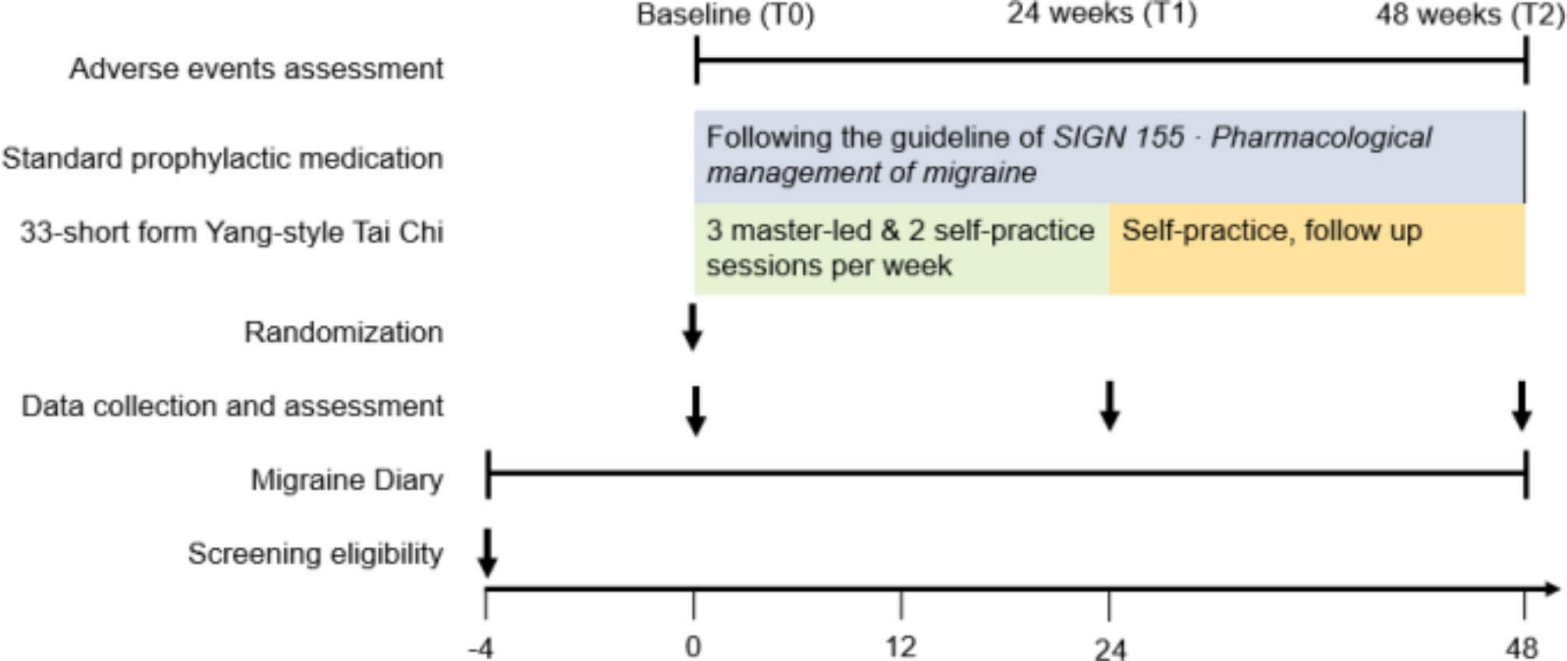
The flow of the study

The present study is a two-arm individual-level non-inferiority randomized controlled trial. A 24-week Tai Chi training with 24-week follow-up will be conducted among Chinese adult women diagnosed with episodic migraine, in comparison to the women receiving standard prophylactic medication. The whole study flow is showed in Fig. 1.

### Participant

The inclusion criteria are: (1) Chinese woman aged 18–65 years. (2) Have a clinical diagnosis of episodic migraine with or without aura according to the International Classification of Headache Disorders, 3rd edition version (ICHD-III) [21]. (3) Experienced migraines with a frequency of three to ten attacks a month. (4) Live in Hong Kong.

The exclusion criteria are: (1) Secondary headache and other neurological disease. (2) More than 5 days of non-migraine headache per month. (3) Experience with Tai Chi or other body-mind exercises (yoga, biofeedback, medication, etc.) after diagnosis of migraine. (4) Drug abuse, take antipsychotic or antidepressant drugs, or take analgesics for other chronic pain more than 3 days a month in the past 12 weeks. (5) Pregnancy or lactation period. (6) Have epilepsy, or psychiatric disease.

### Sample size

The present study, based on the pilot study [13], expect average 3 times (SD: 2.0) and 2 times (SD: 1.5) reduction of migraine attacks after a 24-week Tai Chi intervention and a 24-week follow-up, respectively. A similar clinical efficacy, no difference in migraine attack reduction, is also expected between the Tai Chi group and the prophylactic medication group. According to the Non-inferiority Test of the difference of two means in the PASS software [22], a sample of 88 in each group is required to achieve 95% power to detect non-inferiority between groups at the significant level of 0.05. With the assumption that true difference between the means is 0.00, the margin of non-inferiority is 0.50, and standard deviation is 1.00. With consideration of 20% attrition rate (33/41 = 0.80 in pilot study), 110 subjects (88/0.8 = 110) will be recruited in each group.

A recent RCT study from Best et al. showed that 1-year twice-weekly resistance exercise training with 1-year follow-up could significantly decrease white matter volumes measured in neuroimaging [23]. The present study twice the frequency of Tai Chi exercise compared with Best et al.’s work, which enables a hypothesis that similar result will be achieved after a 24-week Tai Chi intervention and a 24-week follow-up. At least a sample of 4 women is needed to achieve 95% power to detect pre-post variation.

### Recruitment procedure

The research team is conducting an ongoing cohort study which enrolls nearly 4000 Hong Kong women. A database of women willing to participate in any suitable studies has been established during the recruitment process for the cohort study. The recruitment information of the present study will be distributed to these women in the database who are not participating in the previous cohort study. The research team will also distribute emails, flyers and posters containing information of the proposed study to community members living in multiple residential areas. If women have interests to participate the proposed study, they can contact the research team for preliminary registration. A trained research assistant (RA) will preliminarily screen the eligibility during the initial contact. Four weeks further observation will be conducted subsequently, which requires the potential participants to record the migraine attacks through a migraine diary (Fig. 1). The migraine diary is used to record the frequency, intensity, duration, and medications used for migraine attacks. A neurological physician in research team will make migraine diagnosis according to ICHD-III criteria [21] and appraise eligibility of each potential participant based on records from migraine diaries. Those who have between three and ten migraine attacks within four weeks and meet all other eligible criteria will be involved as final participants.

### Baseline assessment and data collection

A structured interview will be conducted to collect information of socio-demographic characteristics, medical history, exercise habits, dietary intake, lifestyle factors (drinking and smoking) and reproductive information from the participants. In addition, participants will be provided with a Migraine Diary [11, 13, 24, 25], and will be instructed to record the details (attack time, length and pain level, etc.) of migraine attack every time from the baseline until the end of the trial (48 weeks). Measurements for major outcomes are stated in the section “Outcome measurements”. All data collection will be conducted at baseline, week 24 and week 48.

### Randomization, concealment and blinding

Eligible participants will be randomly assigned in a 1:1 ratio to either the Tai Chi training group or the standard prophylactic medication group (active control). The computer random number generator will be adopted to perform the randomization using a permuted block algorithm with blocks of size 10. An independent statistician who not involve in the study will conduct the randomization to ensure allocation concealment.

The generated random numbers will be placed in opaque and tamperproof envelopes and sealed. Another research staff will open all envelopes and arrange participants to undertake their corresponding treatments. Participants and investigators will be concealed about the random allocation until the assignments have been made. The RAs who will perform the outcome measurements will be blinded to the treatment group assignment.

### Interventions

#### Tai Chi training

The 24-week Tai Chi training will be prescribed with three 1-hour instructor-led sessions and two 1-hour self-practice sessions per week. Tai chi instructors will be recruited from the Gentle and Tranquil Tai Chi Chuan Association. A modified 33-short form Yang-style Tai Chi Chuan will be adopted as it is the most popular and widely practiced form of Tai Chi in the world. Its short form is typically done with slow, steady movements, which is a practical entry point for many beginners. This short form style of Tai Chi is adopted to reduce the complexity and time required and hence people could learn to practice within a relatively short period of time. The Tai Chi sessions will be operated by qualified/certified instructors. Each 1-hour training session will consist of 10 min brief warm-up stretching session followed by standard Tai Chi routine activities, and 5 min of cool-down stretching. The Tai Chi instructors will have to attend a training session that ensure all of them will deliver the same intervention protocol throughout the study. The RA will monitor the fidelity of intervention by visiting the Tai Chi sessions frequently. The instructor-led sessions will be performed at the University. The training activity will be delivered in group, and the group size is 25–30 persons. During the 24 weeks follow-up period, the participants will be required to practice Tai Chi with the same frequency by themselves.

### Active control: standard prophylactic medication

Participants assigned to the control group will follow the collaborated neurologist’s recommendation to take the prophylactic medication. The Multidisciplinary Panel on Neuropathic Pain (MPNP) of Hong Kong has published the treatment algorithm for migraine, which is in line with the guideline of the Scottish Intercollegiate Guidelines Network (SIGN) on the pharmacological management of migraine (SIGN 155) [26]. Beta-blockers, specifically metoprolol and propranolol, are one of the first-line treatments for migraine prophylaxis. Generally, beta-blockers are well tolerated. It was widely used and the efficacy was compared with non-pharmacological treatment like acupuncture [27] and Yoga [28]. The dosage will start on 10 mg and slowly increase by 10 mg every week until the dosage reached the highest dose of 150 mg/day. The whole course of amitriptyline will last for 48 weeks. The second line drug such as amitriptyline and the third line drug such as gabapentin will be adopted according to doctor’s judgement if necessary. The RA will help the participants to make appointment with the neurological doctor, to obtain the prescription. The adverse events will be recorded accordingly.

### Outcome measurements

#### Primary outcomes

The primary outcome for the efficacy examination will be calculated as the differences of mean number of migraine days and migraine attacks between 4 weeks before randomization and weeks 21–24 / 45–48 after randomization. The number of migraine days and migraine attack will be calculated based on the migraine diary records.

The primary outcome of the mechanism exploration is the changes of WMHs measured by the magnetic resonance imaging (MRI). All the participants in the Tai Chi group will receive MRI scan at baseline to identify those with WMHs, and these participants will receive MRI scan again at 24th weeks and 48th weeks. MRI will be performed at the University Research Facility in Behavioral and Systems Neuroscience (UBSN) in the University. MRI will be performed with a 3.0-Tesla MRI scanner (Siemens Medical System, Erlangen, Germany) and a 32-channel head coil. WMH was visible as hyperintense lesions on T2-weighted and Fluid-Attenuated Inversion Recovery (FLAIR) images, and as isointense or slightly hypointense lesions on T1-weighted images. MRI scan will be used to record the appearance, number, size and location of WMH. The degree of WMH was assessed using the Scheltens visual rating scale [29]. WMH will be separately graded in each of the following locations: frontal lobes, temporal lobes, parietal lobes and occipital lobes. WMH will be graded as follows: 0 (no lesions), 1 (hyperintensity < 3 mm and n ≤ 5), 2 (< 3 mm and n ≥ 6), 3 (4–10 mm and n ≤ 5), 4 (4–10 mm and n ≥ 6), 5 (≥ 11 mm and n ≥ 1), and 6 (confluent). The sum of scores from each location was considered as the final score [30].

### Secondary outcomes

The secondary outcomes include the changes from baseline to 24th week and 48th week of the following assessments.

(1) The proportion of responders.

Responder is defined as at least 50% reduction in the number of migraine attacks per month [27]. The migraine attacks will be recorded in the migraine diary.

(2) The intensity of headache.

Visual Analogue Scale (VAS) will be used to measure headache intensity [31]. VAS requires participants to record pain intensity on a 100 mm length horizontal VAS line, the left end is “no pain” and the right end is “most severe pain imaginable”. The participant will be instructed to draw a vertical line on the horizontal VAS line based on the experience of their own headache.

(3) The duration of headache.

The headache duration will be recorded in the migraine diary to the nearest 0.1 h.

(4) Migraine related disability.

Migraine Disability Assessment Score (MIDAS) will be used to measure migraine related disability [32]. MIDAS contains 5 questions which inquiries about the number of days of missed work/school, reduced productivity at work/school, missed household work, reduced productivity in household work, and missed family and/or social activities. Responses to each question are scaled in units of days and reported as either the number of days missed or reduced productivity. The total MIDAS score is composed of the sum of the 5 items.

(5) Stress level.

The Perceived Stress Scale (PSS) will be adopted to measure stress level [33]. PSS contains 14 items to measure the degree to which participant appraises situations in their lives as stressful during the previous month. The 14-item PSS includes 7 positive items and 7 negative items, each rated on a 5-point Likert scale.

(6) Sleep quality.

The Chinese version of the Pittsburgh Sleep Quality Index will be used to measure sleep quality [34]. PSQI contains 19 items which involves seven components. These seven components include subjective sleep quality, sleep latency, sleep duration, habitual sleep efficiency, sleep disturbances, sleep medication, and daytime dysfunction. The possible score range for each component is 0 to 3, 0 is “no difficulty” and 3 is “severe difficulty”. The total MIDAS score is the sum of the 7 component scores, and a higher score indicates poorer subjective sleep quality.

(7) Fatigue level.

Fatigue Numeric Rating Scale (NRS) will be used to measure fatigue level [35]. NRS is a 11-point horizontal scale anchored at 0 and 10, 0 is “no fatigue” and 10 is “as bad as you can imagine”. Participants will be asked to rate fatigue by selecting the number that describes the worst level of fatigue during the past 24 h.

(8) Quality-of-Life (QOL).

Migraine-Specific Quality-of-Life Questionnaire will be used to measure QOL [36]. MSQ contains 14 items across three dimensions, which includes Role Restrictive (RR), Role Preventive (RP), and Emotional Function (EF). Items are on a standard six-point ordered-categorical scale with choices ranging from none of the time to all of the time. All domains of the MSQ are scored from 0 to 100, and a higher score indicates a better QOL.

(9) The anthropometric variables.

The weight will be measured using a digital weighing scale calibrated in kilograms, and the height will be measured using a digital stadiometer calibrated in meters. The body mass index (BMI) will be calculated by dividing the body weight in kilograms by the square of the height in meters.

The waist circumference will be measured using a measuring tape, which will be measured halfway between the lowest rib and the top of the hipbone, roughly in line with the umbilicus. The hip circumference will be measured using a measuring tape, which will be measured at the largest circumference around the hip. The waist hip ratio (WHR) will be calculated by dividing the waist circumference by the hip circumference.

The percent body fat will be measured using a body composition analyzer according to the manufacturer’s instructions (InBody 270; Biospace Inc., Seoul, Korea). InBody utilizes bioelectrical impedance analysis (BIA) technology to measure human body composition and automatically calculates percent body fat.

(10) Inflammation markers.

Enzyme linked immunosorbent assay (ELISA) will be performed to measure the C-reactive proteins (CRP), Interleukin 6 (IL-6), tumor necrosis factor-alpha (TNF-α), and calcitonin gene-related peptide (CGRP). Blood samples (10 ml) will be drawn from the antecubital vein of participant by a nurse. Serum will be separated after centrifugation for 10 min at 2,000 g and frozen at -20 °C until assay. The serum sample will be used to perform ELISA testing.

(11) Cerebrovascular functions.

The cerebrovascular function indicators involve PI, MBFV, and CVR. Transcranial Doppler (TCD) will be used to measure these cerebrovascular function indicators. MBFV and PI in all intracranial and neck vessels will be recorded. CVR will be calculated by: CVR=△MBFV=(V stim-V rest)/V rest*100, where △MBFV is the relative change of blood flow velocity, V stim is the blood flow velocity during the stimulation, and V rest is the baseline flow velocity during the initial 5 min prior to stimulation. The value will be calculated by the mean of both left and right brain and from 10 cycles [37].

### Assessment of compliance, safety, and report of adverse events

Compliance to the Tai Chi training will be monitored consecutive by session attendance and exercise logs. Instructor-led session attendance ≥ 85% and self-practice session implementation ≥ 75% is defined as optimal compliance. The RA will review participants’ adherence regularly, discuss with them barriers to adherence and encourage compliance. The RAs and Tai Chi masters will monitor the whole exercise process of participants in the Tai Chi training group. The RAs will record the medication information and supervise the entire medication process of participants in the active control group. The participant will be allowed to seek medical support and use acute pain relief medication during an acute migraine attack. The adverse events will be recorded by the RAs and reported to the principal investigator (PI). The PI and co-investigators will review the case and determine whether to stop or continue study for that participant. The event will be reported to the Research Ethics Committee.

### Data analysis

The trained student helps will work in pairs for data entry and double check. The statistical expert in the research team will be responsible for data clean, data coding and analysis. After data clean (handle skewed data, outliers, and missing data), baseline characteristics will be described and compared between groups by One-way ANOVA and Pearson chi-square test for continuous and categorical variables, respectively.

To address the first objective, which examines the clinical efficacy of Tai Chi on migraine prophylaxis and its prolonged effectiveness. First, the 24 weeks and 48 weeks changes of each outcome variable will be calculated in each group. The within-group differences between baseline and 24 weeks, baseline and 48 weeks will be examined by Paired t-test or where appropriate. Then the repeated analyses of covariance (ANCOVA) with adjustment of baseline characteristics will be applied, to examine the between-group differences in terms of primary and secondary outcomes (those for efficacy examination) from baseline to 24 weeks and 48 weeks, respectively. The time × group interaction effects between intervention group and control group will be examined. For the proportion of responders, a chi-square test is applied. Both per protocol analysis and intention-to-treat analysis (ITT) will be implemented. The former will include the participants who complete the 24 weeks Ta Chi training or medication and 24 weeks of follow-up, the latter is for all participants who attend baseline assessment.

To address the second objective, which explores the mechanisms of Tai Chi in the prophylaxis of migraine. First, all the participants in the Tai Chi group will receive the MRI scan, TCD examination and ELISA test. They will receive TCD examination and ELISA test again at 24th week (T_1_) and 48th week (T_2_). While only the participant who was identified WMH (Scheltens visual rating scores > 0) will take the MRI scan again at the T_1_ and T_2_. The prevalence of WMH in migraineurs ranged from 38 to 44% [15], we believe that we can include the enough cases for analysis. The associations between the changes of WMH and migraine features will be analyzed by the Mix model across T_0_, T_1_, and T_2_ time point. Similar analysis will be conducted for the changes of cerebrovascular flow process (PI, MBFV, CVR) and migraine features, as well as for inflammatory markers (serum levels of CRP, CGRP, TNF-a and IL-6) and migraine features before and after Tai Chi training. In addition, as a supplementary analysis, the changes of some potential trigger factors (stress, fatigue, sleep) and their associations with the migraine features will also be analyzed by the Mix model. Statistic software SPSS 26.0 (SPSS Institute) is used for analysis. All statistical tests are two-sided and a p-value < 0.05 is considered statistically significant.

### Ethical considerations

Written informed consent will be obtained from each participant after a verbal and a written explanation (via an information sheet) of the purpose and procedures of the study. Participation in the study will be on a voluntary basis, and all of the potential subjects will be assured that they have the right to refuse or withdraw from the study at any time without penalty. All information related to participants will remain confidential. The information about each participant will be assigned a code known only to the researcher, which will not allow participant to be identified in any research outputs. The data will be stored with encryption in an office computer. Nobody besides the researcher will have access to the data. No personal information will be linked to the data. After completion of the study, all data will be stored for 5 years, and destroyed thereafter. Responsible members of The Hong Kong Polytechnic University may be given access for monitoring and/or auditing of the research.

The study proposal has received ethical approval from the PolyU Institutional Review Board of the Hong Kong Polytechnic University (HSEARS20220721006).

## Discussion

As a category of complementary medicine, traditional Tai Chi has been considered as a holistic approach in promoting rehabilitation and health among different populations [9, 38, 39]. Our pilot study provided preliminary evidence about the prophylaxis effect of Tai Chi on migraine [11, 13], which arouses our interests in further investigating the underlying mechanisms on how Tai Chi works for the improvement of migraine attack. We thereby plan to conduct the proposed study. The key issues we want to address are to identify whether Tai Chi is appropriate as a stand-alone preventive strategy that has comparable treatment efficacy with standard prophylactic medication; and if it is proven to be effective, what mechanism leads to such intervention effect.

To our knowledge, this is the first study using a modified short-form Tai Chi training for the prophylaxis of episodic migraine and comparing its clinical efficacy with the well-established standard prophylactic medication. Given the importance of reducing disease burden and financial cost of migraine attacks, the findings of this study will provide new insights regarding the role of a mind-body aerobic exercise in migraine prophylaxis. It has beneficial effects and advantages brings to the economy, society, public services, health, as well as the individuals’ quality of life and well-being. If it is proven to be effective, it adds new knowledge to non-pharmacological prophylactic treatments for migraine. It is expected that the results of this study would be readily implemented by making evidence-based suggestion to the healthcare and medical practitioners, the health policy-making authorities, and the public. This study will also investigate the mechanism of intervention effect by analyzing the changes and associations of migraine features with neurovascular functions and inflammatory markers during and after the Tai Chi training. Evidence emerged to explore the mechanism of aerobic exercise on migraine prophylaxis, which mainly derived from animal models and observational studies [40]. This study would lead to a better understanding on the mechanisms and pathways in the relationship between exercise and migraine improvement. The possible outcomes of this research project in terms of new knowledge and practical applications include provides evidence that Tai Chi can be used as a new non-pharmacological treatment for migraine prophylaxis, which further demonstrates that Tai Chi can be broadly promoted in public as an alternative therapy for migraine management; and also reveals insight that Tai Chi can exert prophylaxis effects on migraine by improving neurovascular functions, which further lays foundation for future studies in therapeutic potential of Tai Chi on other neurovascular diseases. We believe that the new findings and valuable knowledge got from this groundbreaking research project would contribute a lot to both the academia and public.

## Data Availability

Not applicable.
